# Joint Longitudinal Low Calcium High Phosphorus Trajectory Associates with Accelerated Progression, Acute Coronary Syndrome and Mortality in Chronic Kidney Disease

**DOI:** 10.1038/s41598-020-66577-7

**Published:** 2020-06-15

**Authors:** I-Wen Ting, Hung-Chieh Yeh, Han-Chun Huang, Hsiu-Yin Chiang, Pei-Lun Chu, Chin-Chi Kuo

**Affiliations:** 1 Division of Nephrology, Department of Internal Medicine, China Medical University Hospital and College of Medicine, China Medical University, Taichung, Taiwan; 2 Big Data Center, China Medical University Hospital, China Medical University, Taichung, Taiwan; 3 Department of Internal Medicine, Fu Jen Catholic University Hospital, Fu Jen Catholic University, New Taipei, Taiwan; 4 School of Medicine, College of Medicine, Fu Jen Catholic University, New Taipei, Taiwan

**Keywords:** Kidney, End-stage renal disease

## Abstract

The effects of long-term disturbance of the mineral metabolism on patients with chronic kidney disease (CKD) are unclear. We investigated whether the longitudinal Ca-P (joint calcium and phosphorus) trajectories are associated with incident end-stage renal disease (ESRD), acute coronary syndrome (ACS), and all-cause mortality in patients with CKD. We conducted a prospective cohort study by using data from a 13-year multidisciplinary pre-ESRD care registry. The final study population consisted of 4,237 CKD patients aged 20–90 years with data gathered from 2003 to 2015. Individuals’ Ca-P trajectories were defined using group-based multi-trajectory modeling into three distinct patterns: reference, moderately abnormal, and severely abnormal. Times to ESRD, ACS, and death were analyzed using multiple Cox regression. Compared with those with a “reference” Ca-P trajectory, the adjusted hazard ratios (aHRs) (95% confidence interval [CI]) for incidental ESRD were 5.92 (4.71–7.44) and 15.20 (11.85–19.50) for “moderately abnormal” and “severely abnormal” Ca-P trajectories, respectively. The corresponding aHRs for ACS were 1.94 (1.49–2.52) and 3.18 (2.30–4.39), and for all-cause mortality, they were 1.88 (1.64–2.16) and 2.46 (2.05–2.96) for “moderately abnormal” and “severely abnormal” Ca-P trajectories, respectively. For outcomes of progression to ESRD, the detrimental effects of abnormal Ca-P trajectories were more substantial in patients with CKD stage 3 than those with CKD stage 4 or 5 (*p*-value for interaction < 0.001). Future studies should validate reliable longitudinal cut-offs of serum phosphorus and consider the “lowering phosphorus— the lower the better, the earlier the better” approach to phosphorus control in CKD.

## Introduction

Disturbances in the mineral metabolism and parathyroid hormone (PTH)–vitamin D endocrine loop are prevalent in patients with advanced chronic kidney disease (CKD) and typically leads to bone abnormalities in turnover, mineralization, volume, and strength—which is also known as “CKD-MBD (mineral and bone disorders)”^1,2^. Increasing evidence links CKD-MBD to multiple extra-skeletal complications, including vascular calcification^3–6^, cardiovascular disease (CVD)^7,8^, and cardiovascular (CV)^9–11^ and all-cause mortality^8,10–15^, particularly for common markers of mineral metabolism such as calcium, phosphorus, and PTH. However, most studies have focused on patients with end-stage renal disease (ESRD) requiring renal replacement therapy (RRT) and on the outcome of all-cause mortality^16^. Despite these studies consistently reporting a positive relationship between elevated serum phosphorus level and all-cause mortality, the role of serum calcium level and Ca×P (the multiplication product of calcium and phosphorus) on mortality is less consistent and is not clearly established^16,17^.

For patients with CKD not requiring dialysis, although a recent meta-analysis indicated that higher serum phosphorus levels are associated with rapid progression of CKD toward ESRD and all-cause mortality^18^, available evidence is not conclusive, and this association may be modified by age^19,20^. In the Modification of Diet in Renal Disease (MDRD) Study cohort, serum phosphorus and Ca×P levels were not significantly associated with all-cause or CVD mortality in patients with CKD stages 3 to 4, after adjustment for eGFR^21^. Moreover, the relationship between serum phosphorus and the accelerated progression of CKD was not supported in the Kidney Early Evaluation Program, which was based on a study sample of 10,672 CKD patients^22^.

In the general population, mounting evidence exists showing that slightly abnormal phosphorus level is independently associated with CVD and CV mortality, which implies that the biological effects of phosphorus goes beyond the uremic toxicity^7,23^. Among Framingham Offspring study participants free of CVD and CKD at baseline, higher serum phosphorus levels were associated with increased CVD risk^24^. In the prospective Coronary Artery Risk Development in Young Adults (CARDIA) study, participants with higher serum phosphorus level, even within normal range, had significantly increased coronary calcification measured using CT-based calcium scoring^25^. More research efforts are required to investigate whether higher serum phosphorus levels are predictors of the development of CKD.

Scant studies have used repeat measurements to describe longitudinal trajectories of serum calcium, phosphorus, or Ca×P in the course of CKD to evaluate the influence of these long-term mineral abnormalities on the risk of CKD progression to dialysis and mortality. Only one study of an ESRD population attempted to associate longitudinal changes of mineral parameters, calcium, phosphorus, Ca×P, and PTH with all-cause mortality^15^. In the present study, we conducted a large pre-ESRD registry-based CKD cohort study and took advantage of regular monitoring of mineral metabolism and kidney function based on pre-ESRD care protocol to capture the Ca-P (joint calcium and phosphorus) trajectories. This enabled us to evaluate the associations among mineral metabolism joint trajectories and adverse outcomes of CKD composed of rapid progression to ESRD, acute coronary syndrome (ACS), and all-cause mortality.

## Results

**Figure 1:**
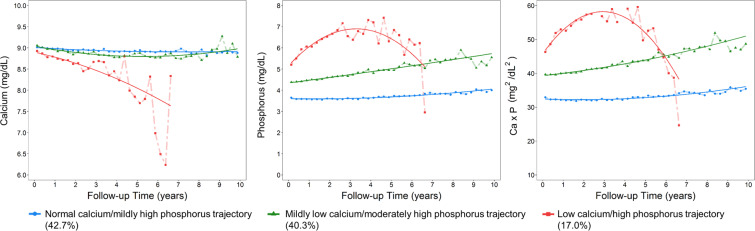
Calcium, phosphors, and Ca×P trajectories defined by group-based multi-trajectory modelling (GBMM) of serial quarterly average levels of calcium, phosphorus, and Ca×P. The solid line is the averaged estimated trajectory, whereas the points represent the averaged observed trajectory (N = 4,237). **Reference Ca-P trajectory**: Normal calcium/ mildly high phosphorus trajectory; **Moderately abnormal Ca-P trajectory**: Mildly low calcium/ moderately high phosphorus trajectory; **Severely abnormal Ca-P trajectory:** Low calcium/ high phosphorus trajectory.

Among the 4,237 participants in the program, the median age at enrollment was 67.5 years (IQR: 57.2–76.0), the median follow-up time was 32.7 months, and the median number of serum calcium and phosphorus measurements was 6 (IQR: 4–12). Three distinct Ca-P trajectories were identified using GBMM: 1) Normal calcium/ mildly high phosphorus trajectory (reference Ca-P trajectory, 42.7%); 2) Mildly low calcium/ moderately high phosphorus trajectory (moderately abnormal Ca-P trajectory, 40.3%) and 3) Low calcium/ high phosphorus trajectory (severely abnormal Ca-P trajectory, 17.0%) (Fig. 1).

Compared with patients with a reference Ca-P trajectory (their longitudinal phosphorus level being stable below 4 mg/dL), patients with abnormal Ca-P trajectories were more likely to be younger at enrollment and female, as well as to have advanced CKD stage 4 and 5, diabetes, and hypertension with correspondingly more frequent use of antidiabetic and antihypertensive medication (Table 1). At enrollment, the median eGFR level significantly decreased across the joint Ca-P trajectories, with results as follows: “reference” (34.0 ml/min/1.73m^2^), “moderately abnormal” (21.3 ml/min/1.73m^2^), and “severely abnormal” (11.2 ml/min/1.73m^2^) (*p* for trend < 0.001). Similarly, the levels of hemoglobin, serum calcium and albumin, and urine creatinine declined across the joint Ca-P trajectories (in the order from reference to severely abnormal); however, the opposite trend was observed for the levels of serum creatinine, phosphorus, uric acid, total cholesterol, triglyceride, protein–creatinine ratio (PCR), iPTH (intact parathyroid hormone), and the utilization of phosphorus binder and vitamin D across the three Ca-P trajectories (in the order from reference to severely abnormal) (Table 1).

**Table 1:** Baseline demographic and clinical characteristics according to CaP trajectories defined by group-based multitrajectory modelling (GBMM).

Variables	Total (N = 4237)	Normal calcium/mildly high phosphorus trajectory (n = 1810)	Mildly low calcium/moderately high phosphorus trajectory (n = 1705)	Low calcium/high phosphorus trajectory (n = 722)	P-value^†^	P for trend^‡^
Age at entry (year), median (IQR)	67.5 (57.2, 76.0)	70.6 (59.9, 78.1)	67.2 (57.6, 75.9)	61.6 (51.2, 70.3)	<0.001	<0.001
Female, n (%)	1918 (45.3)	548 (30.3)	988 (58.0)	382 (52.9)	<0.001	<0.001
Follow-up duration (month), median (IQR)	32.7 (19.5, 56.3)	44.7 (27.0, 72.4)	32.5 (20.6, 50.8)	16.3 (11.0, 24.5)	<0.001	<0.001
No. of Ca, P and Ca × P records, median (IQR)	6 (4, 12)	6 (3, 12)	7 (4, 13)	6 (3, 10)	<0.001	0.988
BMI (kg/m^2^), median (IQR)	24.2 (22.0, 26.9)	24.2 (22.0, 26.7)	24.2 (21.8, 27.0)	24.2 (21.9, 27.0)	0.970	0.813
Initial CKD stage, n (%)					<0.001	-
1	64 (1.5)	36 (2.0)	27 (1.6)	1 (0.1)		
2	137 (3.2)	85 (4.7)	44 (2.6)	8 (1.1)		
3	1561 (36.9)	1011 (55.9)	496 (29.2)	54 (7.5)		
4	1386 (32.8)	526 (29.1)	668 (39.3)	192 (26.7)		
5	1079 (25.5)	150 (8.3)	464 (27.3)	465 (64.6)		
Smoking, n (%)					0.017	-
Never	3553 (83.9)	1484 (82.0)	1457 (85.5)	612 (84.8)		
Former	291 (6.9)	151 (8.3)	98 (5.8)	42 (5.8)		
Current	393 (9.3)	175 (9.7)	150 (8.8)	68 (9.4)		
Alcohol consumption, n (%)					0.043	-
Never	3872 (91.4)	1629 (90.0)	1582 (92.8)	661 (91.6)		
Former	223 (5.3)	109 (6.0)	73 (4.3)	41 (5.7)		
Current	142 (3.4)	72 (4.0)	50 (2.9)	20 (2.8)		
Education level (year), n (%)					<0.001	-
<9	1071 (25.3)	421 (23.3)	472 (27.7)	178 (24.7)		
9 ≤ ~ <12	1670 (39.4)	672 (37.1)	704 (41.3)	294 (40.7)		
12 ≤ ~ <16	1008 (23.8)	445 (24.6)	374 (21.9)	189 (26.2)		
16+	488 (11.5)	272 (15.0)	155 (9.1)	61 (8.5)		
Diabetes, n (%)	1495 (35.4)	489 (27.1)	670 (39.3)	336 (46.8)	<0.001	<0.001
Hypertension, n (%)	2438 (57.7)	952 (52.7)	1025 (60.2)	461 (64.2)	<0.001	<0.001
Cardiovascular disease, n (%)	1393 (33.0)	625 (34.6)	558 (32.8)	210 (29.3)	0.035	0.011
**Baseline medication profiles, n (%)**						
Pentoxifylline	1176 (28.4)	535 (30.3)	474 (28.3)	167 (23.7)	0.004	0.001
NSAIDs	971 (23.4)	464 (26.3)	376 (22.4)	131 (18.6)	0.0001	<0.001
Contrast media	274 (6.6)	124 (7.0)	109 (6.5)	41 (5.8)	0.537	0.266
***Anti-platelet***						
Dipyridamole	313 (7.6)	149 (8.4)	123 (7.3)	41 (5.8)	0.076	0.024
Aspirin, Ticlopidine, Clopidogrel	1026 (24.8)	478 (27.1)	391 (23.3)	157 (22.3)	0.010	0.004
***Anti-hypertension agents***						
ACEI	836 (20.2)	334 (18.9)	358 (21.4)	144 (20.4)	0.201	0.207
ARBs	1793 (43.3)	702 (39.8)	764 (45.6)	327 (46.4)	0.001	<0.001
Collapse (Trichlormethiazide, Furosemide, Spironolactone, Amizide, Indapamide)	1977 (47.7)	687 (38.9)	850 (50.7)	440 (62.4)	<0.001	<0.001
***Anti-diaetes agents***						
OAD	1203 (29.0)	414 (23.5)	546 (32.6)	243 (34.5)	<0.001	<0.001
Insulin	808 (19.5)	237 (13.4)	377 (22.5)	194 (27.5)	<0.001	<0.001
***Anti-lipidemic agents***						
Statin	994 (24.0)	396 (22.4)	404 (24.1)	194 (27.5)	0.028	0.009
Fibrate	244 (5.9)	93 (5.3)	97 (5.8)	54 (7.7)	0.073	0.034
***P-binder***						
Aluminum	3 (0.1)	0 (0.0)	2 (0.1)	1 (0.1)	0.362	0.161
Calcium	782 (18.9)	181 (10.3)	345 (20.6)	256 (36.3)	<0.001	<0.001
Vitamin D	59 (1.4)	14 (0.8)	30 (1.8)	15 (2.1)	0.011	0.004
***Calcium-based phosphorus binders utilization trajectory***						
Seldom	2227 (52.6)	1456 (80.4)	714 (41.9)	57 (7.9)	<0.001	-
Occasionally to often	970 (22.9)	207 (11.4)	529 (31.0)	234 (32.4)		
Frequently	1040 (24.6)	147 (8.1)	462 (27.1)	431 (59.7)		
***Elemental Calcium trajectory***						
Very low / none	2285 (53.9)	1475 (81.5)	745 (43.7)	65 (9.0)	<0.001	-
Low	989 (23.3)	222 (12.3)	531 (31.1)	236 (32.7)		
Moderate	963 (22.7)	113 (6.2)	429 (25.2)	421 (58.3)		
**Baseline biochemical profiles, median (IQR)**						
Ca (mg/dL)	8.90 (8.50, 9.20)	9.00 (8.60, 9.30)	8.90 (8.50, 9.20)	8.60 (8.20, 9.00)	<0.001	<0.001
Ca adjusted by albumin (mg/dL)	9.02 (8.72, 9.32)	9.00 (8.72, 9.30)	9.06 (8.76, 9.34)	8.98 (8.62, 9.30)	<0.001	0.452
P (mg/dL)	4.10 (3.60, 4.70)	3.60 (3.20, 4.00)	4.30 (3.90, 4.80)	5.00 (4.40, 5.70)	<0.001	<0.001
Ca x P (mg^2^/dL^2^)	37.2 (32.2, 42.7)	32.5 (29.0, 36.3)	39.1 (35.2, 43.3)	44.7 (39.5, 50.5)	<0.001	<0.001
eGFR (mL/min/1.73m^2^)	24.6 (13.7, 38.3)	34.0 (23.5, 44.5)	21.3 (13.1, 33.6)	11.2 (7.5, 18.2)	<0.001	<0.001
Hemoglobin (g/dL)	10.7 (9.3, 12.4)	11.9 (10.3, 13.5)	10.4 (9.1, 11.8)	9.6 (8.5, 10.7)	<0.001	<0.001
Serum creatinine (mg/dL)	2.32 (1.64, 3.75)	1.83 (1.50, 2.50)	2.50 (1.75, 3.84)	4.58 (3.13, 6.22)	<0.001	<0.001
Serum uric acid (mg/dL)	7.40 (6.30, 8.70)	7.20 (6.10, 8.40)	7.50 (6.30, 8.80)	7.90 (6.70, 9.20)	<0.001	<0.001
Serum albumin (g/dL)	3.90 (3.40, 4.20)	4.00 (3.60, 4.30)	3.80 (3.40, 4.20)	3.50 (3.10, 4.00)	<0.001	<0.001
Sodium(mmol/L)	138 (136, 140)	138 (136, 140)	138 (136, 140)	138 (135, 140)	0.000	<0.001
Potassium (mmol/L)	4.30 (3.90, 4.70)	4.20 (3.80, 4.60)	4.30 (3.90, 4.80)	4.40 (3.90, 5.00)	<0.001	<0.001
High-density lipoprotein (HDL) (mg/dL)	40.3 (33.9, 49.0)	39.7 (33.3, 49.0)	40.5 (34.3, 49.0)	40.8 (34.1, 49.0)	0.548	0.317
Low-density lipoprotein (LDL) (mg/dL)	105 (83, 129)	105 (85, 126)	104 (81, 130)	109 (84, 138)	0.070	0.059
Triglyceride (TG) (mg/dL)	132 (91, 193)	127 (88, 180)	135 (90, 200)	141 (102, 221)	<0.001	<0.001
Total cholesterol (T-CHO) (mg/dL)	184 (156, 215)	181 (155, 210)	184 (156, 216)	190 (159, 233)	<0.001	<0.001
TG/HDL ratio	3.56 (2.23, 5.94)	3.51 (2.15, 5.45)	3.59 (2.24, 6.17)	3.65 (2.35, 6.45)	0.055	0.016
Urine creatinine (mg/dL)	79.0 (52.6, 114.3)	94.2 (62.6, 139.6)	73.6 (49.9, 103.3)	64.3 (45.9, 88.5)	<0.001	<0.001
Intact-PTH (pg/mL)	155 (60, 299)	71 (38, 161)	141 (57, 242)	256 (102, 404)	<0.001	<0.001
Urine protein-to-creatinine ratio (mg/g cre)	1054 (330, 2648)	420 (147, 1167)	1346 (595, 3171)	2968 (1410, 5926)	<0.001	<0.001

^†^P-values are calculated by Kruskal-Wallis test for continuous variables and Chi-square test for categorical variables.

^‡^P-values for trend are calculated by Spearman’s correlation for continuous variables and by Cochran-Armitage trend test for binary variables.

**Abbreviations:** Ca: calcium, Ca x P: calcium-phosphate product, CKD: chronic kidney disease, eGFR: estimated glomerular filtration rate, GBMM: group-based multitrajectory modelling, IQR: inter-quartile range.

**Figure 2:**
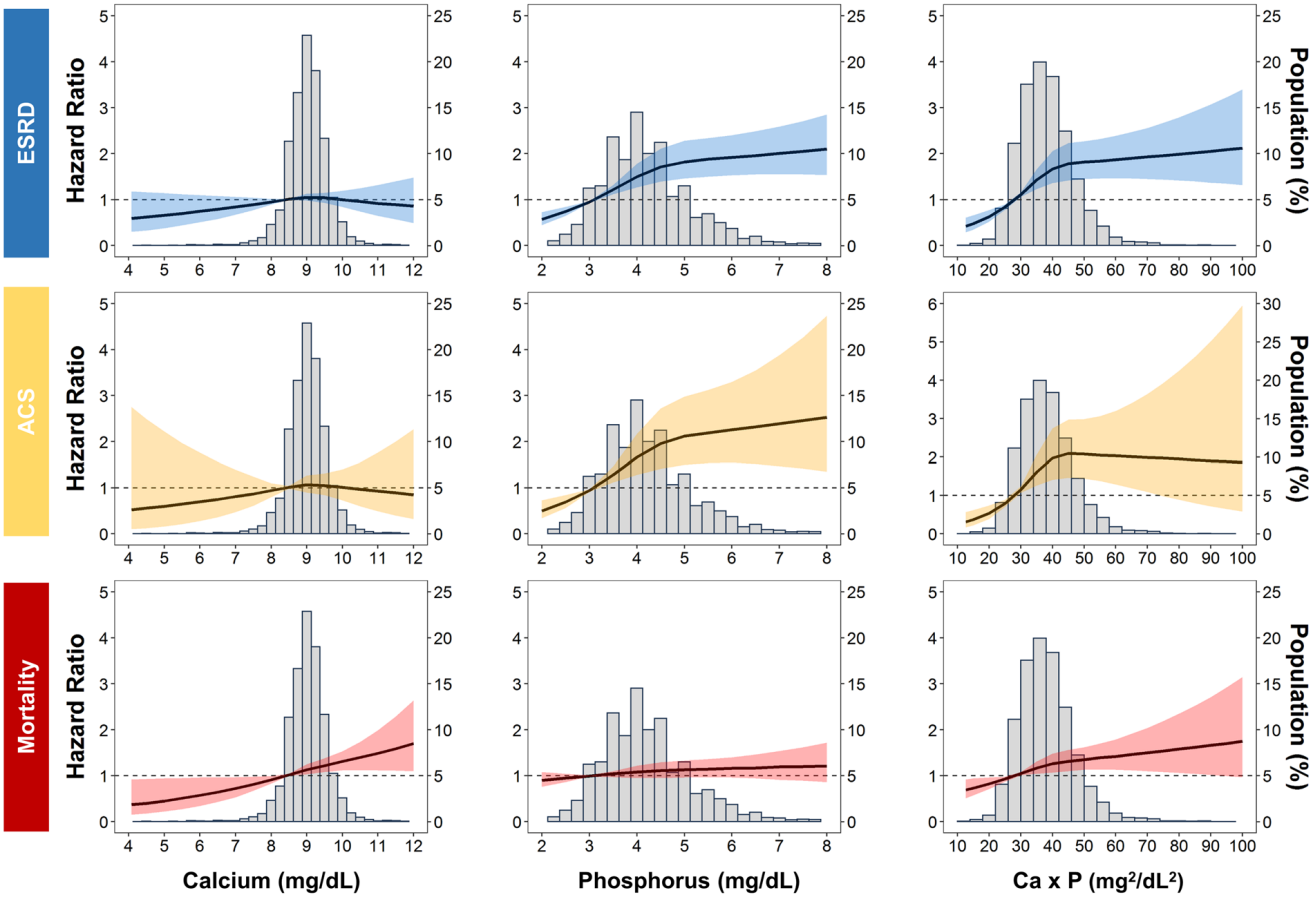
Adjusted hazard ratios (HRs) for end-stage renal disease (ESRD) requiring dialysis, Acute coronary syndrome (ACS) and all-cause mortality according to the baseline levels of calcium, phosphorus, and calcium-phosphorus (Ca×P) product. Solid lines represent adjusted HRs based on restricted cubic splines for baseline calcium, phosphorus and Ca×P, with knots at the 10th, 50th, and 90th percentiles. Shaded areas represent upper and lower 95% confidence intervals. Reference was set at 10th percentile of baseline calcium, phosphorus and Ca×P. Upper panel: risk of progression to ESRD requiring dialysis (blue); middle panel: risk of ACS (orange); lower panel: all-cause mortality (red). Variables adjusted are the same as those shown in Model 3 in Table 2.

**Figure 3:**
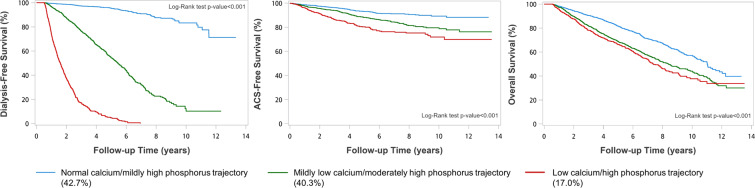
Kaplan-Meier curves of dialysis-free survival, ACS-free survival and overall survival, according to Ca-P trajectories generated by group-based multi-trajectory modelling (N = 4237). ACS, Acute coronary syndrome.

**Figure 4:**
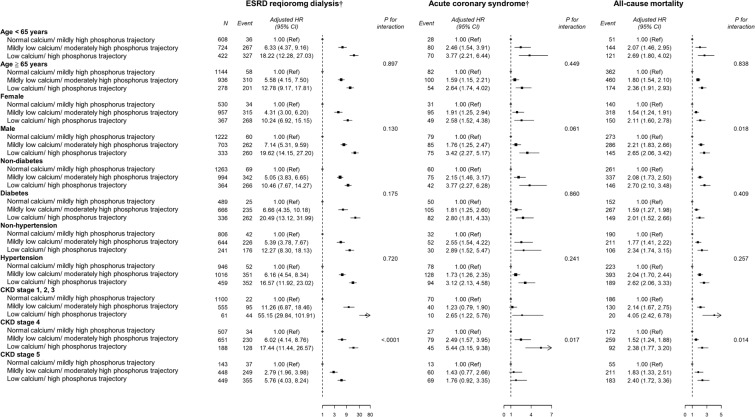
Subgroup analysis for associations between Ca-P trajectories and adverse outcomes according to baseline characteristics. Reference Ca-P trajectory: Normal calcium/ mildly high phosphorus trajectory; **Moderately abnormal Ca-P trajectory**: Mildly low calcium/ moderately high phosphorus trajectory; **Severely abnormal Ca-P trajectory:** Low calcium/ high phosphorus trajectory.

A total of 1,234 ESRD events and 1,340 deaths occurred during the 14,577.1 person-years of follow-up. Incidental rates of ESRD, ACS, and all-cause mortality were 84.6, 21.4, and 64.7 per 1000 person-years, respectively. Baseline levels of phosphorus and Ca×P were linearly associated with the accelerated CKD progression to ESRD. By contrast, it was baseline calcium and Ca×P levels that were linearly associated all-cause mortality (Fig. 2). However, increased risk of ACS associated only with baseline phosphorus concentration (Fig. 2). Kaplan–Meier curves showed that participants with a “severely abnormal” Ca-P trajectory presented much shorter dialysis-free survival, ACS-free survival, and overall survival (log-rank test, *P* < 0.001) (Fig. 3). Compared with those with a “reference” Ca-P trajectory, the adjusted hazard ratio (aHR) (95% confidence interval [CI]) for incidental ESRD was 5.92 (4.71–7.44) and 15.20 (11.85–19.50) in “moderately abnormal” and “severely abnormal” trajectories, respectively (Table 2**, Model 3**). The corresponding aHR (95% CI) for ACS was 1.94 (1.49–2.52) and 3.18 (2.30–4.39) respectively for “moderately abnormal” and “severely abnormal” trajectories. For all-cause mortality, it was 1.88 (1.64–2.16) and 2.46 (2.05–2.96) respectively for “moderately abnormal” and “severely abnormal” trajectories (Table 2**, Model 3**). The statistical inferences remained the same in coarsened exact matching (CEM) analyses although effect sizes for different clinical outcomes were slightly attenuated (Supplementary Tables 1 and 2). In the subgroup analysis, male CKD patients had significantly higher risk of all-cause mortality across the Ca-P trajectories compared to those of female patients (*p*-value for interaction=0.018) (Fig. 4). For outcomes of progression to ESRD, the detrimental effects of abnormal Ca-P trajectories were more substantial in patients with CKD stage 3 than those with CKD stage 4 or 5 (*p*-value for interaction <0.001). However, the opposite was observed for the outcome of ACS (Fig. 4).

**Table 2:** Adjusted hazard ratios (95% confidence interval) of risk of progression to end-stage renal disease (ESRD), acute coronary syndrome (ACS,) and all-cause mortality by Ca-P trajectories.

	N	Cases	Person-years	Incidence	Crude HR (95% CI)	Model 1	Model 2	Model 3
						Adjusted HR (95% CI)	Adjusted HR (95% CI)	Adjusted HR (95% CI)
**ESRD requiring dialysis**^†^								
Normal calcium/mildly high phosphorus trajectory	1810	99	7960.05	12.4	1.00 (Ref)	1.00 (Ref)	1.00 (Ref)	1.00 (Ref)
Mildly low calcium/moderately high phosphorus trajectory	1705	594	5435.19	109.3	8.84 (7.14, 10.94)	9.50 (7.63, 11.82)	5.98 (4.77, 7.50)	5.92 (4.71, 7.44)
Low calcium/high phosphorus trajectory	722	541	1181.87	457.7	36.74 (29.54, 45.69)	39.15 (31.31, 48.95)	15.68 (12.27, 20.04)	15.20 (11.85, 19.50)
*P* for trend					<0.001	<0.001	<0.001	<0.001
**Acute coronary syndrome**^†^								
Normal calcium/mildly high phosphorus trajectory	1810	113	8881.55	12.7	1.00 (Ref)	1.00 (Ref)	1.00 (Ref)	1.00 (Ref)
Mildly low calcium/moderately high phosphorus trajectory	1705	183	7684.90	23.8	2.01 (1.59, 2.55)	2.29 (1.80, 2.93)	1.92 (1.48, 2.48)	1.94 (1.49, 2.52)
Low calcium/high phosphorus trajectory	722	126	3183.48	39.6	3.75 (2.88, 4.88)	4.28 (3.26, 5.63)	3.26 (2.37, 4.49)	3.18 (2.30, 4.39)
*P* for trend					<0.001	<0.001	<0.001	<0.001
**All-cause mortality**								
Normal calcium/mildly high phosphorus trajectory	1810	425	9150.44	46.4	1.00 (Ref)	1.00 (Ref)	1.00 (Ref)	1.00 (Ref)
Mildly low calcium/moderately high phosphorus trajectory	1705	611	8065.62	75.8	2.08 (1.83, 2.36)	2.25 (1.97, 2.56)	1.88 (1.64, 2.16)	1.88 (1.64, 2.16)
Low calcium/high phosphorus trajectory	722	304	3493.91	87.0	3.38 (2.90, 3.95)	3.54 (3.02, 4.15)	2.53 (2.12, 3.04)	2.46 (2.05, 2.96)
*P* for trend					<0.001	<0.001	<0.001	<0.001

Incidence = No. of incident dialysis cases/person-years*1000.

^†^With competing risk analysis for death.

**Model 1:** Adjusted for gender, BMI, smoking status, alcohol consumption, education (n = 4210).

**Model 2:** Adjusted for gender, BMI, smoking status, alcohol consumption, education, diabetes, hypertension, cardiovascular disease, primary etiologies of CKD, and baseline eGFR (n = 4193).

**Model 3:** Adjusted for gender, BMI, smoking status, alcohol consumption, education, diabetes, hypertension, cardiovascular disease, primary etiologies of CKD, baseline eGFR, and profiles of baseline medication (n = 4112).

**Abbreviations:** BMI: body mass index, Ca: calcium, Ca × P: calcium-phosphate product, CI: confidence interval, CKD: chronic kidney disease, eGFR: estimated glomerular filtration rate, ESRD: end stage renal disease, HR: hazard ratio, P: phosphorus.

**Reference Ca-P trajectory**: Normal calcium/ mildly high phosphorus trajectory; **Moderately abnormal Ca-P trajectory**: Mildly low calcium/ moderately high phosphorus trajectory; **Severely abnormal Ca-P trajectory:** Low calcium/ high phosphorus trajectory.

## Discussion

By using GBMM to model the trajectory of parameters of mineral metabolism, composed of serum calcium, phosphorus, and Ca×P, jointly among patients with CKD in the pre-ESRD program, we identified three distinct joint trajectories. We observed that a severely abnormal Ca-P trajectory was an independent risk factor of progression to ESRD, developing ACS, and all-cause mortality in the CKD population. Our results implied that a strict control of mineral metabolism for maintaining the longitudinal phosphorus level stably below 4 mg/dL is a potential therapeutic target for halting CKD progression, particularly in male patients and patients with CKD stage 3.

Most studies of ESRD populations supported that hyperphosphatemia and higher Ca×P levels increased the risk of all-cause death although different cut-off levels were suggested^16,26–28^. Correspondingly, existing guidelines did not specify the target range of serum phosphorus and simply suggested to keep serum phosphorus to “normal range” with low confidence of evidence quality^13,29^. In 2002 KDOQI guideline, hyperphosphatemia was defined as serum phosphorus level >4.5 mg/dL^30^. Yet, prior studies evaluated the cut-off point of serum phosphorus at a much higher level. For example, in a study that used two national, random, prevalent samples of hemodialysis patients, baseline serum phosphorus levels of >6.5 mg/dL had a 27% higher mortality risk (RR: 1.27) than did patients with phosphorus levels of 2.4–6.5 mg/dL; patients with high Ca×P levels (**>**72 mg^2^/dL^2^) had a mortality risk of 1.34 relative to those with products of 42–52 mg^2^/dL^2^^14^. Data from 40,538 hemodialysis patients showed that the risk of death increased when serum phosphorus levels were >5.0 mg/dL^27^. The relative risk (RR) of death for Ca×P levels of 50–55 mg^2^/dl^2^ was 1.14 (1.05–1.23), compared with when values were 40–45 mg^2^/dL^2^ ^27^. Ganesh *et al*. showed that strong associations existed between elevated serum phosphorus (>6.5 vs. 2.4–6.5 mg/dL) and Ca×P levels (RR 1.06 for each 10 mg^2^/dL^2^ increase in Ca×P) with CV mortality in HD patients, especially mortality caused by CAD and sudden death^9^. Macro *et al*. also showed that there was an increased risk of CV death in patients with serum phosphorus levels of >6.5 mg/dL or Ca×P levels of >52 mg^2^ /dl^2 ^^31^. In United States Renal Data System (USRDS) study of 14,829 HD patients, serum phosphorus, calcium, and Ca×P were associated with fatal and nonfatal CV events^32^. The inference based on evidence from general population or CKD populations was more heterogeneous; however, the critical cut-off of serum phosphorus levels were closer to 4.5 mg/dL, which is more aligned with our proposed therapeutic targets – longitudinal phosphorus level stably below 4 mg/dL. Despite the lack of significant associations between serum phosphorus and all-cause or CVD mortality after adjustment for GFR in the MDRD cohort, other studies have supported the detrimental role of serum phosphorus (>4.3 vs. <3.3 mg/dL) and Ca×P (>40 vs. <30 mg^2^/dL^2^)^21,33,34^.

The underlying pathophysiology explaining why abnormal Ca-P trajectories are associated with CKD progression may be partially explained by hyperphosphatemia or the “precipitation–calcification hypothesis”: excess phosphorus in the kidney produces precipitation and deposition of calcium phosphorus microcrystals in the renal tubular lumen, capillaries, and interstitium and results in progressive functional deterioration in chronic renal failure^35^. Animal models of CKD have supported this hypothesis by showing that calcium–phosphorous crystals can evoke an inflammatory response leading to fibrosis, loss of nephrons, and eventually to chronic renal failure^36^. Moreover, in uremic rat models, the group treated with sevelamer, a noncalcium-based phosphorus binder, had less renal calcium phosphorus deposition and tubulointerstitial fibrosis and better preservation of renal function when compared with those treated with calcium carbonate^37^. Our study provided the first large longitudinal clinical evidence to support the independent role of Ca-P trajectories in accelerating renal failure, particularly in patients with CKD stage 3, implying the crucial window for early serum phosphorus control in CKD care.

The mechanisms of abnormal Ca-P trajectories related to ACS and all-cause mortality have been thought to be mainly caused by soft tissue metastatic calcification, especially vascular calcification, in patients with chronic renal failure^5,38^. In addition to Ca-P deposition on vessels, “ossification” of vascular smooth muscle cells (VSMCs) caused by hyperphosphatemia has also been demonstrated to contribute to vascular calcification^5,38,39^. Early animal studies have reported that increased Ca×P was associated with soft tissue metastatic calcification^40,41^. Similarly, Ca×P was reported to have a strong positive correlation with myocardial calcification in ESRD patients^42^. In a uremic rat model, high dietary phosphorus levels and hyperphosphatemia both induced cardiac fibrosis and arterial wall thickening^43^. More recent studies have demonstrated that calcium and phosphorus directly affect VSMCs that promote vascular calcification^5^. Elevated phosphorus levels affect multiple signaling pathways that increase the susceptibility of VSMCs to calcification including decreased calcification inhibitors, increased extracellular matrix (ECM) degradation, osteo/chondrogenic differentiation, apoptosis, and vesicle release^5^. Furthermore, the effects of elevated calcium and phosphorus levels are synergistic, providing a major stimulus for vascular calcification in CKD^5,44^.

Ours was the first study using GBMM to evaluate the joint effects of serial serum calcium, phosphorus, and Ca×P on adverse outcomes in patients with CKD. Many related studies have supported that elevated phosphorus and Ca×P levels are associated with an increased risk of morbidity and mortality; however, most of them have been based on a single measurement. Thus, these studies may suffer from potential misclassification bias and from losing integrative information about the dynamics of mineral metabolism through the CKD course. For example, only repeated measurements of serum calcium and phosphorus levels can reflect the summation of dysregulation of calcium/phosphorus homeostasis over the course of CKD (e.g., the activation of FGF23–αKlotho endocrine axis), dietary intervention, and the use of a phosphorus binder^45^. Only one study attempted to analyze the effects of longitudinal change of mineral metabolism parameters on mortality in patients with CKD^15^. When Melamed *et al*. used baseline data to perform their analysis, no association between Ca×P and mortality was observed. However, when time-dependent Ca×P values were used, the highest quartile of Ca×P (>56.4 mg^2^/dl^2^) was associated with an 87% higher risk of mortality compared with the reference quartile (40.2–47.7 mg^2^/dl^2^)^46^. In our study, we observed that abnormal Ca-P trajectories increased the risk of all-cause death. The downwards bending curve observed among patients with a low calcium / high phosphorus trajectory reflects the real-world clinical practice and its corresponding response rate (Fig. 1). In this group, the average eGFR was about 11 ml/min/1.73m^2^ that have significantly reduced the survival time to ESRD. Those who had longer survival time prior to ESRD may have better controlled calcium and phosphorus homeostasis due to more intensive medical intervention and better self-dietary control; however, the numbers of such patients were not many causing a much larger trajectory fluctuation in the end of the curve. Our study is the first to echo the latest Kidney Disease: Improving Global Outcomes (KDIGO) clinical practice guidelines recommending that in patients with CKD G3a**–**G5D, treatments of CKD-MBD should be based on serial assessments of phosphorus, calcium, and iPTH levels all considered together, not just based on a single point measurement^47^. Also, our findings signified the requirement to evaluate the longitudinal effectiveness of phosphorus binders, used for treatment of hyperphosphatemia, in reducing the risk of ESRD, ACS, and mortality in CKD populations.

The finding showed that male CKD patients are more vulnerable to abnormal calcium and phosphorus homeostasis regarding the outcome of all-cause mortality and acute coronary syndrome (ACS) is practically important. Although the biological explanation for this vulnerability remains unclear, one recent paper also showed that a strong inverse association between serum phosphorus level and the heat-induced skin hyperemic response, a marker of microvascular function, was only observed in men, but not in women (*p*-value for interaction was 0.01)^48^. Their findings partially explain our results from a mechanistic perspective. Whether sex-specific cutoffs for serial phosphorus level among CKD patients should be conducted in daily practice requires further research.

The strength of this study lies in the care taken to minimize exposure misclassification by modeling Ca-P trajectories. We also sought to mediate the confounding effects of kidney function by controlling baseline eGFR and primary etiologies concomitantly. More relevantly, we used CEM to ensure the comparability of baseline eGFR across two main joint trajectory patterns as well as that the statistical inferences and effects sizes were similar. Nonetheless, this study had several limitations. First, the information of phosphorus binder adherence was not available. This prevented us from evaluating the effectiveness of phosphorus binders. Second, the concern of residual confounding cannot be completely resolved in a nonrandomized study using registry-based EMRs. For example, information such as genetic data, dietary patterns, and environmental factors (e.g., air pollution and metal exposure) were not considered in this study. Third, missing data was a critical challenge in this longitudinal study. Although frequent measurements of serum calcium and phosphorus over a long-term follow-up period (median of 6 measurements) minimized the concern, the informative drop-out (i.e., patients having fewer Ca-P measurements due to suffering an event) remains an insufficiently addressed source of bias. The sequential modeling framework did not directly propagate parameter uncertainty from the GBMM to Cox proportional hazards modeling, which may lead to biased estimates^49^. However, we also performed joint latent class modeling to confirm the robustness of our approach and the inferences stayed the same (data not shown). Fourth, this study was performed at a single tertiary medical center, which may limit the generalizability of our findings. Nonetheless, biochemical and pathophysiological similarities across different populations enhance the external validity of our findings.

In conclusion, our study demonstrated that the abnormal Ca-P trajectories are independently associated with accelerated kidney failure, ACS, and all-cause mortality in patients with CKD, even after adjustment for the use of phosphorus binders. Minimizing derangement of the mineral metabolism, potentially targeting at the longitudinal phosphorus level stably below 4 mg/dL, is critical to stabilize the progression of CKD and prevent complications. Future studies should verify reliable cut-offs of serum phosphorus and consider “lowering phosphorus – the lower the better, the earlier the better” approach to phosphorus control in CKD.

## Methods

### Study population

**Figure 5:**
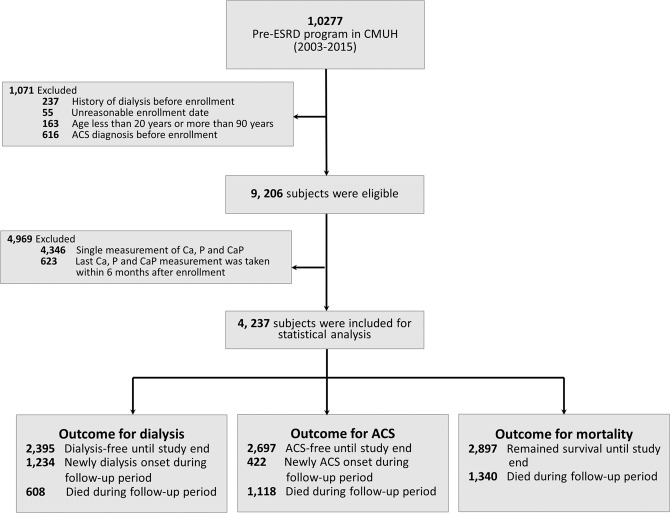
Flow diagram of the study selection process.

Detailed description of the China Medical University Hospital (CMUH) pre-ESRD registry and national dataset tracking has been published recently^50^. For more than a decade, CMUH has been using EMRs to facilitate care management. It also established the CMUH Clinical Research Data Repository (CRDR) in 2017, which carefully verifies and validates data from various clinical sources to unify trackable patient information generated during the health care process^50^. This has made it possible to integrate data related to the CMUH pre-ESRD Program with the CMUH CRDR, which contains data on laboratory tests, medications, special procedures, and admission records. Participants aged 20–90 years who had their serum calcium and phosphorus levels measured at least twice over a period of at least 6 months were then selected from this kidney disease-centered integrative database. The index date was defined as the first day of enrollment in the pre-ESRD program. Patients were required to have undergone at least 6 months of continuous post enrollment observation to enable a sufficient number of follow-ups required for observation of the outcomes of interest. We excluded patients aged less than 20 or over 90 years (n = 163), those who had undergone dialysis (n = 237) and has had ACS before enrollment (n = 616), cases with illogical data entries (n = 55), and those who did not undergo a sufficient number of serum calcium and phosphorus measurements (n = 4,969). This left a total of 4,237 participants in the present study (Fig. 5). The comparison of sociodemographic and clinical characteristics between excluded and included patients was provided in the Supplementary Table 3. The study was approved by the Big Data Center of China Medical University Hospital and the Research Ethical Committee/Institutional Review Board of China Medical University Hospital (CMUH105-REC3–068) and the need to obtain informed consent for the present study was waived by the Research Ethical Committee of China Medical University Hospital.

### Measurements of serum calcium and phosphorus levels and of kidney function

Serum calcium and phosphorus levels were measured using the timed endpoint colorimetric method. Creatinine levels were measured using the Jaffe rate method (kinetic alkaline picrate) with a Beckman UniCel® DxC 800 (Beckman Coulter Inc., CA, USA) at the CMUH Central Laboratory. The eGFR levels were estimated using the abbreviated Chronic Kidney Disease Epidemiology Collaboration (CKD-EPI) equation (eGFR = 141 × min(Scr/κ, 1)^α^ × max(Scr/κ, 1)^−1.209^ × 0.993^Age^ × 1.018 [if female] × 1.159 [if black])^51^. Serum creatinine levels at enrollment were used to define the baseline eGFR level and corresponding CKD stages by using the following cut-off values:>90, 60–89.9, 30–59.9, 15–29.9, and <15 ml/min/1.73 m^2^. All prospective serum calcium, phosphorus, and eGFR measurements (until the end-points) were taken into account. We calculated the quarterly average level of serum calcium and phosphorus in cases where the patient had more than one serum calcium and phosphorus measurement within a 3-month period. The trajectory of serum calcium, phosphorus, and Ca×P for each individual was modeled based on these serial quarterly average measurements (Fig. 1). Random spot urine dipstick and PCR measurements were used to quantify proteinuria. Proteinuria was defined as PCR ≥ 200 mg/g from random spot samples. Detailed information of other covariables was described in **Supplementary method**.

### Statistical analysis

Continuous variables were expressed as median and interquartile range (IQR) values and compared using the Kruskal–Wallis test. Categorical variables were expressed as a frequency (percentage) and compared using the chi-square test. We then performed a two-stage analysis to reveal the relationships of Ca-P joint trajectories, ESRD risk, ACS, and all-cause mortality.

#### Stage 1: Identification of distinctive Ca-P trajectories

The semiparametric group-based multitrajectory modeling (GBMM), a generalization of the basic univariate group-based trajectory modeling (GBTM)^52–54^, was used to characterize longitudinal joint trajectories of serum calcium, phosphorus, and Ca×P among patients enrolled in the CMUH Pre-ESRD Program throughout the follow-up period. Briefly, GBMM fits a finite mixture model to identify latent clusters of patients following similar trajectories of multiple parameters of mineral metabolism (SAS TRAJ macro)^55^. For trajectories of the utilization of calcium-based phosphorus binders and estimated elemental calcium amount from calcium-based phosphorus binders, we applied the original GBTM (Supplementary Fig. 1). Detailed statistical procedures of GBTM are described in our previous papers^50,56^. The number of subgroup assignments of Ca-P trajectories was determined by balancing clinical knowledge against latent hypotheses regarding the existence of distinct trajectories to facilitate meaningful interpretation. The determination of Ca-P trajectories was performed prior to analysis pertaining to the risk of dialysis, ACS, and mortality. Missing rates of covariables were summarized in Supplementary Table 4.

#### Stage 2: Association analysis between Ca-P trajectories and adverse outcomes

We evaluated the prospective relationships between Ca-P trajectories and the risks of progressing to dialysis, ACS, and mortality by using hazard ratios (HRs) with a 95% CI based on the Cox proportional hazards model. The models were adjusted hierarchically (see footnotes of Table 2). To account for the fact that renal function is a critical confounding factor pertaining to the influence of Ca-P trajectories on the risk of progression to dialysis, ACS, and death, we formulated multiple domains of renal function composed of baseline eGFR, and primary etiologies of CKD within a multivariable model to minimize residual confounding. The dose-response relationship of each Ca-P trajectory was evaluated by examining HRs across trajectory groups, with significance being evaluated by testing groups of trajectories as a continuous variable. We characterized the relationship between Ca-P trajectories and the risk of progressing to dialysis or ACS by competing risk analysis using both cause-specific models (Table 2) and the methods outlined by Fine and Gray (Supplementary Table 5) with the aim of minimizing potential bias introduced by a competing risk of death^57^.

Exploratory subgroup analysis was used to evaluate effect modification in the adjusted models. Patients were stratified according to (1) age (65+ vs. <65), (2) gender, (3) presence of diabetes, and (4) presence of hypertension. Several sensitivity analyses were conducted to evaluate the robustness of our findings (Supplementary Table 6). First, adjusting the baseline PCR levels and proteinuria status in the fully adjusted model was shown not to alter the risk of progression to dialysis or mortality, despite a considerable reduction in the sample size. Second, we took serum albumin measurements into account as an additional adjustment for baseline levels, yet still obtained stable results (Supplementary Table 6). Third, to control the most critical confounding factor, baseline eGFR, we additionally applied a CEM analysis with matching criteria of age, sex, and baseline kidney function to specifically adjust for imbalanced kidney function among three joint trajectories (in this analysis, we collapsed three trajectories into two trajectories [reference vs. abnormal] to maintain sample size) followed by statistical adjustment for residual confounders in the stratified Cox regression modeling (Supplementary Tables 1 and 2). All sensitivity analyses showed results consistent with the primary analysis. All statistical analyses were performed using SAS, version 9.4 (SAS Institute Inc., Cary, NC, USA). The two-sided statistical significance level was set at α = 0.05.

### Ethical approval

The study was approved by the Research Ethical Committee/Institutional Review Board of China Medical University Hospital (CMUH105-REC3-068).

## Supplementary information

Supplementary material.

## Data Availability

The data that support the findings of this study are available on request from the corresponding author, CCK. The data are not publicly available due to them containing information that could compromise research participant privacy.

## References

[1] Cozzolino M (2014). Is chronic kidney disease-mineral bone disorder (CKD-MBD) really a syndrome?. Nephrology, dialysis, transplantation: official publication of the European Dialysis and Transplant Association - European Renal Association.

[2] Moe S (2006). Definition, evaluation, and classification of renal osteodystrophy: a position statement from Kidney Disease: Improving Global Outcomes (KDIGO). Kidney Int.

[3] Goodman WG (2000). Coronary-artery calcification in young adults with end-stage renal disease who are undergoing dialysis. N. Engl. J. Med..

[4] Qunibi, W. Y., Nolan, C. A. & Ayus, J. C. Cardiovascular calcification in patients with end-stage renal disease: a century-old phenomenon. *Kidney Int Suppl*, S73–80, 10.1046/j.1523-1755.62.s82.15.x (2002). 10.1046/j.1523-1755.62.s82.15.x 12410860

[5] Shanahan CM, Crouthamel MH, Kapustin A, Giachelli CM (2011). Arterial calcification in chronic kidney disease: key roles for calcium and phosphate. Circulation research.

[6] Schlieper G, Schurgers L, Brandenburg V, Reutelingsperger C, Floege J (2016). Vascular calcification in chronic kidney disease: an update. Nephrology, dialysis, transplantation: official publication of the European Dialysis and Transplant Association - European Renal Association.

[7] Heine GH, Nangaku M, Fliser D (2013). Calcium and phosphate impact cardiovascular risk. European heart journal.

[8] Slinin Y, Foley RN, Collins AJ (2005). Calcium, phosphorus, parathyroid hormone, and cardiovascular disease in hemodialysis patients: the USRDS waves 1, 3, and 4 study. J Am Soc Nephrol.

[9] Ganesh SK, Stack AG, Levin NW, Hulbert-Shearon T, Port FK (2001). Association of elevated serum PO(4), Ca x PO(4) product, and parathyroid hormone with cardiac mortality risk in chronic hemodialysis patients. J Am Soc Nephrol.

[10] Block GA (2004). Mineral metabolism, mortality, and morbidity in maintenance hemodialysis. J Am Soc Nephrol.

[11] Tentori F (2008). Mortality risk for dialysis patients with different levels of serum calcium, phosphorus, and PTH: the Dialysis Outcomes and Practice Patterns Study (DOPPS). Am J Kidney Dis.

[12] Kestenbaum B (2005). Serum phosphate levels and mortality risk among people with chronic kidney disease. J Am Soc Nephrol.

[13] Kidney Disease: Improving Global Outcomes (KDIGO) CKD–MBD Work Group. KDIGO clinical practice guideline for the diagnosis, evaluation, prevention, and treatment of Chronic Kidney Disease-Mineral and Bone Disorder (CKD-MBD). *Kidney Int Suppl*, S1–130, 10.1038/ki.2009.188 (2009). 10.1038/ki.2009.188 19644521

[14] Block GA, Hulbert-Shearon TE, Levin NW, Port FK (1998). Association of serum phosphorus and calcium x phosphate product with mortality risk in chronic hemodialysis patients: a national study. Am J Kidney Dis.

[15] Melamed ML (2006). Changes in serum calcium, phosphate, and PTH and the risk of death in incident dialysis patients: a longitudinal study. Kidney Int.

[16] Natoli JL (2013). Is there an association between elevated or low serum levels of phosphorus, parathyroid hormone, and calcium and mortality in patients with end stage renal disease? A meta-analysis. BMC nephrology.

[17] Palmer SC (2011). Serum levels of phosphorus, parathyroid hormone, and calcium and risks of death and cardiovascular disease in individuals with chronic kidney disease: a systematic review and meta-analysis. JAMA.

[18] Da J (2015). Serum Phosphorus and Progression of CKD and Mortality: A Meta-analysis of Cohort Studies. Am J Kidney Dis.

[19] Toapanta Gaibor NG, Nava Perez NC, Martinez Echevers Y, Montes Delgado R, Guerrero Riscos MA (2017). PTH levels and not serum phosphorus levels are a predictor of the progression of kidney disease in elderly patients with advanced chronic kidney disease. Nefrologia: publicacion oficial de la Sociedad Espanola Nefrologia.

[20] Chang AR, Anderson C (2017). Dietary Phosphorus Intake and the Kidney. Annual review of nutrition.

[21] Menon V (2005). Relationship of phosphorus and calcium-phosphorus product with mortality in CKD. Am J Kidney Dis.

[22] Mehrotra R (2013). No independent association of serum phosphorus with risk for death or progression to end-stage renal disease in a large screen for chronic kidney disease. Kidney Int.

[23] Ketteler M, Wolf M, Hahn K, Ritz E (2013). Phosphate: a novel cardiovascular risk factor. European heart journal.

[24] Dhingra R (2007). Relations of serum phosphorus and calcium levels to the incidence of cardiovascular disease in the community. Arch Intern Med.

[25] Foley RN, Collins AJ, Herzog CA, Ishani A, Kalra PA (2009). Serum phosphorus levels associate with coronary atherosclerosis in young adults. J Am Soc Nephrol.

[26] Block GA, Hulbert-Shearon TE, Levin NW, Port FK (1998). Association of serum phosphorus and calcium x phosphate product with mortality risk in chronic hemodialysis patients: a national study. Am J Kidney Dis.

[27] Block GA (2004). Mineral metabolism, mortality, and morbidity in maintenance hemodialysis. J Am Soc Nephrol.

[28] Tentori F (2008). Mortality risk for dialysis patients with different levels of serum calcium, phosphorus, and PTH: the Dialysis Outcomes and Practice Patterns Study (DOPPS). Am J Kidney Dis.

[29] Kidney Disease: Improving Global Outcomes (KDIGO) CKD-MBD Update Work Group. KDIGO 2017 Clinical Practice Guideline Update for the Diagnosis, Evaluation, Prevention, and Treatment of Chronic Kidney Disease–Mineral and Bone Disorder (CKD-MBD). *Kidney Int Suppl*. **7** (2017). 10.1016/j.kisu.2017.10.001 PMC6341011 30681074

[30] National Kidney, F. K/DOQI clinical practice guidelines for chronic kidney disease: evaluation, classification, and stratification. *Am J Kidney Dis* **39**, S1–266 (2002). 11904577

[31] Marco, M. P. *et al*. Higher impact of mineral metabolism on cardiovascular mortality in a European hemodialysis population. *Kidney Int Suppl*, S111–114, 10.1046/j.1523-1755.63.s85.26.x (2003). 10.1046/j.1523-1755.63.s85.26.x 12753279

[32] Slinin Y, Foley RN, Collins AJ (2005). Calcium, phosphorus, parathyroid hormone, and cardiovascular disease in hemodialysis patients: the USRDS waves 1, 3, and 4 study. J Am Soc Nephrol.

[33] Schwarz S, Trivedi BK, Kalantar-Zadeh K, Kovesdy CP (2006). Association of disorders in mineral metabolism with progression of chronic kidney disease. Clinical journal of the American Society of Nephrology: CJASN.

[34] Voormolen N (2007). High plasma phosphate as a risk factor for decline in renal function and mortality in pre-dialysis patients. Nephrology, dialysis, transplantation: official publication of the European Dialysis and Transplant Association - European Renal Association.

[35] Lau K (1989). Phosphate excess and progressive renal failure: the precipitation-calcification hypothesis. Kidney Int.

[36] Khan SR (2004). Crystal-induced inflammation of the kidneys: results from human studies, animal models, and tissue-culture studies. Clinical and experimental nephrology.

[37] Cozzolino M (2002). The effects of sevelamer hydrochloride and calcium carbonate on kidney calcification in uremic rats. J Am Soc Nephrol.

[38] Cozzolino M, Dusso AS, Slatopolsky E (2001). Role of calcium-phosphate product and bone-associated proteins on vascular calcification in renal failure. J Am Soc Nephrol.

[39] Jakoby MG (2000). t. & Semenkovich, C. F. The role of osteoprogenitors in vascular calcification. Current opinion in nephrology and hypertension.

[40] Velentzas C (1978). Visceral calcification and the CaXP product. Advances in experimental medicine and biology.

[41] Alfrey, A. C. The role of abnormal phosphorus metabolism in the progression of chronic kidney disease and metastatic calcification. *Kidney Int Suppl*, S13–17, 10.1111/j.1523-1755.2004.09003.x (2004). 10.1111/j.1523-1755.2004.09003.x 15296502

[42] Rostand SG, Sanders C, Kirk KA, Rutsky EA, Fraser RG (1988). Myocardial calcification and cardiac dysfunction in chronic renal failure. The American journal of medicine.

[43] Amann K (2003). Hyperphosphatemia aggravates cardiac fibrosis and microvascular disease in experimental uremia. Kidney Int.

[44] Reynolds JL (2004). Human vascular smooth muscle cells undergo vesicle-mediated calcification in response to changes in extracellular calcium and phosphate concentrations: a potential mechanism for accelerated vascular calcification in ESRD. J Am Soc Nephrol.

[45] Kuro, O. M. Klotho and endocrine fibroblast growth factors: marker of chronic kidney disease progression and cardiovascular complications? *Nephrology, dialysis, transplantation: official publication of the European Dialysis and Transplant Association - European Renal Association*, 10.1093/ndt/gfy126 (2018). 10.1093/ndt/gfy126 29800324

[46] Melamed ML (2006). Changes in serum calcium, phosphate, and PTH and the risk of death in incident dialysis patients: a longitudinal study. Kidney Int.

[47] Ketteler M (2018). Diagnosis, Evaluation, Prevention, and Treatment of Chronic Kidney Disease-Mineral and Bone Disorder: Synopsis of the Kidney Disease: Improving Global Outcomes 2017 Clinical Practice Guideline Update. Annals of internal medicine.

[48] Ginsberg C (2019). Serum Phosphate and Microvascular Function in a Population-Based Cohort. Clinical journal of the American Society of Nephrology: CJASN.

[49] Sweeting MJ, Thompson SG (2011). Joint modelling of longitudinal and time-to-event data with application to predicting abdominal aortic aneurysm growth and rupture. Biom J.

[50] Tsai CW (2018). Uric acid predicts adverse outcomes in chronic kidney disease: a novel insight from trajectory analyses. Nephrology, dialysis, transplantation: official publication of the European Dialysis and Transplant Association - European Renal Association.

[51] Levey AS (2006). Using standardized serum creatinine values in the modification of diet in renal disease study equation for estimating glomerular filtration rate. Annals of internal medicine.

[52] Nagin DS, Lynam D, Raudenbush S, Roeder K (1999). Analyzing Developmental Trajectories: A Semiparametric, Group-Based Approach. Psychol Methods.

[53] Nagin DS, Odgers CL (2010). Group-based trajectory modeling in clinical research. Annual review of clinical psychology.

[54] Jones BL, Nagin DS, Roeder K (2001). A SAS Procedure Based on Mixture Models for Estimating Developmental Trajectories. Sociological Methods Reserach.

[55] Nagin DS, Jones BL, Passos VL, Tremblay RE (2018). Group-based multi-trajectory modeling. Statistical methods in medical research.

[56] Tsai CW (2018). Longitudinal progression trajectory of random urine creatinine as a novel predictor of ESRD among patients with CKD. Clinica chimica acta; international journal of clinical chemistry.

[57] Fine JP, Gray RJ (1999). A Proportional Hazards Model for the Subdistribution of a Competing Risk. Journal of the American Statistical Association.

