# Supplementary material for: Joint Longitudinal Low Calcium High Phosphorus Trajectory Associates with Accelerated Progression, Acute Coronary Syndrome and Mortality in Chronic Kidney Disease
Source: Sci Rep. 2020 Jun 15;10:9682. doi: 10.1038/s41598-020-66577-7 (PMC7296014; doi:10.1038/s41598-020-66577-7)
Supplement: Supplementary file 1 — Supplementary material. [file 41598_2020_66577_MOESM1_ESM.docx]

**Supplementary Material**

**Title:** Joint Longitudinal Low Calcium High Phosphorus Trajectory Associates with Accelerated Progression, Acute Coronary Syndrome and Mortality in Chronic Kidney Disease

**Authors:** I-Wen Ting, Hung-Chieh Yeh, Han-Chun Huang, Hsiu-Yin Chiang, Pei-Lun Chu, Chin-Chi Kuo

**Supplementary Method**

**Supplementary Table 1.** Baseline characteristic comparison before and after coarsened exact matching (CEM).

**Supplementary Table 2.** Comparison of adjusted hazards ratios based on original and CEM dataset.

**Supplementary Table 3.** Comparison of baseline demographic and clinical characteristics between excluded and included populations.

**Supplementary Table 4.** Missing rate of covariables according to the Ca-P trajectories.

**Supplementary Table 5.** Adjusted hazard ratios (95% confidence interval) of risk of progression to end-stage renal disease (ESRD), acute coronary syndrome (ACS,) and all-cause mortality by Ca-P trajectories (Fine and Gray model). **Reference Ca-P trajectory**: Normal calcium/ mildly high phosphorus trajectory; **Moderately abnormal Ca-P trajectory**: Mildly low calcium/ moderately high phosphorus trajectory; **Severely abnormal Ca-P** **trajectory:** Low calcium/ high phosphorus trajectory.

**Supplementary Table 6.** Adjusted hazard ratios (95% confidence interval [CI]) for the risk of progression to end-stage renal disease (ESRD), acute coronary syndrome, and all-cause mortality when further adjusting for the utilization trajectory of calcium-based phosphorus binders, the trajectory of elemental calcium amount from calcium-based phosphorus binders, baseline protein-to-creatinine ratio (PCR), proteinuria, and serum albumin level.

**Supplementary Figure 1.** Probability of using calcium-based phosphorus binders and quarterly sum of estimated elemental calcium amount (g) from calcium-based phosphorus binders trajectories as defined by group-based trajectory modelling (GBTM). The solid line is the averaged estimated trajectory, whereas the points represent the averaged observed trajectory. (N = 4237). The amount of elemental calcium for calcium carbonate 500 mg and calcium acetate 677 mg is assumed to be 200 mg and 169 mg, respectively.

**Supplementary Method**

***Other variables***

Sociodemographic variables collected during the enrollment interview included age, sex, education, cigarette smoking status, and alcohol consumption. Smoking status was categorized as never, former, and current. Alcohol consumption was categorized as never (< 12 drinks in any 1 year in life), former (≥ 12 drinks in any 1 year and not drinking now), and current (≥12 drinks in any 1 year and currently drinking). Body mass index (BMI) was calculated as the patient’s weight in kilograms divided by their height in meters squared. Registry data or information obtained from EMRs within a 1-year window prior to enrollment was used to compile baseline comorbidities, relevant biochemical measures, and medication use including urate-lowering, glucose lowering, and anti-hypertensive agents. Indications of diabetes mellitus and hypertension were based on the clinical diagnosis of physicians using patient ICD codes and the use of glucose-lowering /anti-hypertensive agents. A history of cardiovascular disease (CVD) was defined as coronary artery disease, myocardial infarction, stroke, or heart failure documented in EMRs.

**Supplementary Table 1.** Baseline characteristic comparison before and after coarsened exact matching (CEM).

|  | **Normal calcium/**  **mildly high phosphorus trajectory** | **Mildly low calcium/moderately**  **high phosphorus trajectory +**  **Low calcium/high phosphorus trajectory** | **P-value** |
| --- | --- | --- | --- |
| **Original Data, n** | 1810 | 2427 |  |
| Age at entry (year), median (IQR) | 70.57 (59.95, 78.09) | 65.42 (55.41, 74.30) | <0.001 |
| Gender, n (%) |  |  | <0.001 |
| Female | 548 (30.28) | 1370 (56.45) |  |
| Male | 1262 (69.72) | 1057 (43.55) |  |
| eGFR (mL/min/1.73m^2^) | 33.98 (23.49, 44.51) | 17.94 (10.45, 29.57) | <0.001 |
| **CEM Data, n** | 1074 | 1074 |  |
| Age at entry (year), median (IQR) | 69.92 (59.88, 77.37) | 69.58 (59.33, 77.26) | 0.676 |
| Gender, n (%) |  |  | 1.000 |
| Female | 467 (43.48) | 467 (43.48) |  |
| Male | 607 (56.52) | 607 (56.52) |  |
| eGFR (mL/min/1.73m^2^) | 27.35 (18.29, 38.26) | 26.83 (17.26, 37.77) | 0.331 |

P-values are calculated by Wilcoxon rank sum test for continuous variables and chi-square test for categorical variables

Multivariate L_1_ distance of the original population: L_1_=0.544; matched population: L_1_=0.346.

**Supplementary Table 2.** Comparison of adjusted hazards ratios based on original and CEM dataset.

|  |  | **ESRD requiring dialysis†** | |  | **Acute coronary syndrome†** | |  | **All-cause mortality** | |
| --- | --- | --- | --- | --- | --- | --- | --- | --- | --- |
|  | **N** | **Event** | **Model 3 HR (95% CI)** |  | **Event** | **Model 3 HR (95% CI)** |  | **Event** | **Model 3 HR (95% CI)** |
| **Original Data** |  |  |  |  |  |  |  |  |  |
| Normal calcium/mildly high phosphorus trajectory | 1810 | 99 | 1.00 (Ref) |  | 113 | 1.00 (Ref) |  | 425 | 1.00 (Ref) |
| Mildly low calcium/moderately high phosphorus trajectory  + Low calcium/high phosphorus trajectory | 2427 | 1135 | 6.88 (5.50, 8.60) |  | 309 | 2.12 (1.64, 2.73) |  | 915 | 1.97 (1.72, 2.25) |
| **CEM Data** |  |  |  |  |  |  |  |  |  |
| Normal calcium/mildly high phosphorus trajectory | 1074 | 81 | 1.00 (Ref) |  | 68 | 1.00 (Ref) |  | 280 | 1.00 (Ref) |
| Mildly low calcium/moderately high phosphorus trajectory  + Low calcium/high phosphorus trajectory | 1074 | 382 | 6.21 (4.83, 7.98) |  | 121 | 1.90 (1.38, 2.60) |  | 413 | 1.88 (1.61, 2.21) |

† With competing risk analysis for death

Original Model 3: Adjusted for gender, BMI, smoking status, alcohol consumption, education, diabetes, hypertension, cardiovascular disease, primary etiologies of CKD, baseline eGFR, and profiles of baseline medication (n=4112)

CEM Model 4: Adjusted for BMI, smoking status, alcohol consumption, education, diabetes, hypertension, cardiovascular disease, primary etiologies of CKD, and profiles of baseline medication (n=2099)

**Abbreviations:** BMI: body mass index, CEM: coarsened exact matching, CI: confidence interval, CKD: chronic kidney disease, eGFR: estimated glomerular filtration rate, ESRD: end stage renal disease, HR: hazard ratio.

**Supplementary Table 3.** Comparison of baseline demographic and clinical characteristics between excluded and included populations.

| **Variables** | **Excluded population (N = 4969)** | **Included population (N = 4237)** | **P-value†** |
| --- | --- | --- | --- |
| Age at entry (year), median (IQR) | 66.3 (55.6, 76.0) | 67.5 (57.2, 76.0) | 0.002 |
| Female, n (%) | 2136 (43.0) | 1918 (45.3) | 0.028 |
| Follow-up duration (month), median (IQR) | 16.3 (6.6, 26.7) | 32.7 (19.5, 56.3) | <.0001 |
| BMI (kg/m^2^), median (IQR) | 24.2 (22.0, 27.1) | 24.2 (22.0, 26.9) | 0.373 |
| Initial CKD stage, n (%) |  |  |  |
| 1 | 310 (6.2) | 64 (1.5) | <.0001 |
| 2 | 477 (9.6) | 137 (3.2) |  |
| 3 | 2018 (40.6) | 1561 (36.9) |  |
| 4 | 885 (17.8) | 1386 (32.8) |  |
| 5 | 1277 (25.7) | 1079 (25.5) |  |
| Smoking, n (%) |  |  |  |
| Never | 4119 (82.9) | 3553 (83.9) | 0.039 |
| Quit | 314 (6.3) | 291 (6.9) |  |
| Yes | 536 (10.8) | 393 (9.3) |  |
| Alcohol consumption, n (%) |  |  |  |
| Never | 4556 (91.7) | 3872 (91.4) | 0.245 |
| Quit | 229 (4.6) | 223 (5.3) |  |
| Yes | 184 (3.7) | 142 (3.4) |  |
| Education level (year), n (%) |  |  |  |
| < 9 | 1402 (28.2) | 1071 (25.3) | 0.015 |
| 9 ≤ ~ <12 | 1905 (38.3) | 1670 (39.4) |  |
| 12 ≤ ~ <16 | 1109 (22.3) | 1008 (23.8) |  |
| 16+ | 553 (11.1) | 488 (11.5) |  |
| Diabetes, n (%) | 1551 (31.4) | 1495 (35.4) | <.0001 |
| Hypertension, n (%) | 2499 (50.6) | 2438 (57.7) | <.0001 |
| Cardiovascular disease, n (%) | 1483 (30.0) | 1393 (33.0) | 0.003 |
| Baseline medication profiles, n (%) |  |  |  |
| Pentoxifylline | 968 (20.4) | 1176 (28.4) | <.0001 |
| NSAIDs | 1184 (25.0) | 971 (23.4) | 0.090 |
| Contrast media | 575 (12.1) | 274 (6.6) | <.0001 |
| *Anti-platelet* |  |  |  |
| Dipyridamole | 242 (5.1) | 313 (7.6) | <.0001 |
| Aspirin, Ticlopidine, Clopidogrel | 1085 (22.9) | 1026 (24.8) | 0.039 |
| *Anti-hypertension agents* |  |  |  |
| ACEI | 894 (18.9) | 836 (20.2) | 0.118 |
| ARBs | 1687 (35.6) | 1793 (43.3) | <.0001 |
| Collapse (Trichlormethiazide, Furosemide,  Spironolactone, Amizide, Indapamide) | 2166 (45.7) | 1977 (47.7) | 0.057 |
| *Anti-diaetes agents* |  |  |  |
| OAD | 1285 (27.1) | 1203 (29.0) | 0.044 |
| Insulin | 777 (16.4) | 808 (19.5) | 0.000 |
| *Anti-lipidemic agents* |  |  |  |
| Statin | 933 (19.7) | 994 (24.0) | <.0001 |
| Fibrate | 220 (4.6) | 244 (5.9) | 0.008 |
| *P-binder* |  |  |  |
| Aluminum | 10 (0.2) | 3 (0.1) | 0.088 |
| Calcium | 831 (17.5) | 782 (18.9) | 0.102 |
| VitaminD | 42 (0.9) | 59 (1.4) | 0.017 |
| Baseline biochemical profiles, median (IQR) |  |  |  |
| Ca (mg/dL) | 8.70 (8.20, 9.10) | 8.90 (8.50, 9.20) | <.0001 |
| Ca adjusted by albumin (mg/dL) | 8.96 (8.64, 9.28) | 9.02 (8.72, 9.32) | <.0001 |
| P (mg/dL) | 4.20 (3.60, 5.30) | 4.10 (3.60, 4.70) | <.0001 |
| Ca x P (mg^2^/dL^2^) | 38.6 (32.5, 47.5) | 37.2 (32.2, 42.7) | <.0001 |
| eGFR (mL/min/1.73m^2^) | 34.9 (13.1, 55.6) | 24.6 (13.7, 38.3) | <.0001 |
| Hemoglobin (g/dL) | 11.0 (9.2, 13.0) | 10.7 (9.3, 12.4) | 0.000 |
| Serum creatinine (mg/dL) | 1.74 (1.25, 3.88) | 2.32 (1.64, 3.75) | <.0001 |
| Serum uric acid (mg/dL) | 7.30 (6.10, 8.60) | 7.40 (6.30, 8.70) | 0.001 |
| Serum albumin (g/dL) | 3.80 (3.20, 4.20) | 3.90 (3.40, 4.20) | <.0001 |
| Na (mmol/L) | 138 (135, 140) | 138 (136, 140) | <.0001 |
| K (mmol/L) | 4.20 (3.80, 4.60) | 4.30 (3.90, 4.70) | <.0001 |
| HDL (mg/dL) | 40.2 (33.8, 48.9) | 40.3 (33.9, 49.0) | 0.857 |
| LDL (mg/dL) | 105 (83, 129) | 105 (83, 129) | 0.822 |
| TG (mg/dL) | 128 (88, 187) | 132 (91, 193) | 0.011 |
| T-CHO (mg/dL) | 181 (154, 212) | 184 (156, 215) | 0.016 |
| TG/HDL ratio | 3.38 (2.03, 5.49) | 3.56 (2.23, 5.94) | 0.014 |
| Urine creatinine (mg/dL) | 81.8 (51.6, 127.4) | 79.0 (52.6, 114.3) | 0.005 |
| Intact-PTH (pg/mL) | 239 (106, 450) | 155 (60, 299) | <.0001 |
| Urine PCR (mg/g) | 629 (162, 2788) | 1054 (330, 2648) | <.0001 |

† P-values are calculated by Kruskal-Wallis test for continuous variables and Chi-square test for categorical variables

**Supplementary Table 4.** Missing rate of covariables according to the Ca-P trajectories.

| Variables | **Total**  **(N = 4237)** | **Normal calcium/mildly high phosphorus trajectory**  **(n = 1810)** | **Mildly low calcium/moderately high phosphorus trajectory**  **(n =1705)** | **Low calcium/high phosphorus trajectory**  **(n = 722)** |
| --- | --- | --- | --- | --- |
| BMI (kg/m2) | 27 | 12 | 10 | 5 |
| Initial CKD stage | 10 | 2 | 6 | 2 |
| Primary Disease | 5 | 1 | 4 | 0 |
| Diabetes | 9 | 4 | 1 | 4 |
| Hypertension | 9 | 4 | 1 | 4 |
| Cardiovascular disease | 9 | 4 | 1 | 4 |
| Medication | 91 | 45 | 29 | 17 |
| Biochemical Profiles |  |  |  |  |
| Ca (mg/dL) | 491 | 302 | 150 | 39 |
| Ca adjusted by albumin (mg/dL) | 752 | 426 | 252 | 74 |
| P (mg/dL) | 611 | 362 | 195 | 54 |
| Ca x P (mg2/dL2) | 881 | 480 | 308 | 93 |
| eGFR (mL/min/1.73m2) | 13 | 5 | 4 | 4 |
| Hemoglobin (g/dL) | 762 | 389 | 284 | 89 |
| Serum creatinine (mg/dL) | 13 | 5 | 4 | 4 |
| Serum uric acid (mg/dL) | 286 | 132 | 111 | 43 |
| Serum albumin (g/dL) | 354 | 196 | 127 | 31 |
| Sodium(mmol/L) | 445 | 236 | 151 | 58 |
| Potassium (mmol/L) | 233 | 120 | 84 | 29 |
| High-density lipoprotein (HDL) (mg/dL) | 2707 | 1231 | 1043 | 433 |
| Low-density lipoprotein (LDL) (mg/dL) | 2056 | 869 | 813 | 374 |
| Triglyceride (TG) (mg/dL) | 376 | 180 | 136 | 60 |
| Total cholesterol (T-CHO) (mg/dL) | 566 | 268 | 210 | 88 |
| TG/HDL ratio | 2777 | 1255 | 1071 | 451 |
| Urine creatinine (mg/dL) | 719 | 342 | 269 | 108 |
| Intact-PTH (pg/mL) | 3941 | 1755 | 1570 | 616 |
| Urine protein-to-creatinine ratio (mg/g cre) | 962 | 444 | 370 | 148 |

Abbreviations: Ca: calcium, Ca x P: calcium-phosphate product, eGFR: estimated glomerular filtration rate, GBMM: group-based multitrajectory modelling.

**Supplementary Table 5.** Adjusted hazard ratios (95% confidence interval) of risk of progression to end-stage renal disease (ESRD), acute coronary syndrome (ACS,) and all-cause mortality by Ca-P trajectories.(Fine and Gray model) . **Reference Ca-P trajectory**: Normal calcium/ mildly high phosphorus trajectory; **Moderately abnormal Ca-P trajectory**: Mildly low calcium/ moderately high phosphorus trajectory; **Severely abnormal Ca-P** **trajectory:** Low calcium/ high phosphorus trajectory.

|  |  |  |  |  |  | **Model 1** | **Model 2** | **Model 3** |
| --- | --- | --- | --- | --- | --- | --- | --- | --- |
|  | **N** | **Cases** | **Person-years** | **Incidence** | **Crude HR**  **(95% CI)** | **Adjusted HR**  **(95% CI)** | **Adjusted HR**  **(95% CI)** | **Adjusted HR**  **(95% CI)** |
| **ESRD requiring dialysis**† |  |  |  |  |  |  |  |  |
| Normal calcium/mildly high phosphorus trajectory | 1810 | 99 | 7960.05 | 12.4 | 1.00 (Ref) | 1.00 (Ref) | 1.00 (Ref) | 1.00 (Ref) |
| Mildly low calcium/moderately high phosphorus trajectory | 1705 | 594 | 5435.19 | 109.3 | 8.00 (6.49, 9.85) | 8.22 (6.63, 10.19) | 5.16 (4.12, 6.46) | 5.14 (4.10, 6.45) |
| Low calcium/high phosphorus trajectory | 722 | 541 | 1181.87 | 457.7 | 29.66 (23.92, 36.78) | 30.47 (24.38, 38.09) | 12.42 (9.66, 15.98) | 12.26 (9.51, 15.80) |
| *P* for trend |  |  |  |  | <0.001 | <0.001 | <0.001 | <0.001 |
| **Acute coronary syndrome**† |  |  |  |  |  |  |  |  |
| Normal calcium/mildly high phosphorus trajectory | 1810 | 113 | 8881.55 | 12.7 | 1.00 (Ref) | 1.00 (Ref) | 1.00 (Ref) | 1.00 (Ref) |
| Mildly low calcium/moderately high phosphorus trajectory | 1705 | 183 | 7684.90 | 23.8 | 1.65 (1.30, 2.09) | 1.81 (1.42, 2.31) | 1.53 (1.18, 1.98) | 1.57 (1.21, 2.04) |
| Low calcium/high phosphorus trajectory | 722 | 126 | 3183.48 | 39.6 | 2.56 (1.97, 3.32) | 2.80 (2.14, 3.68) | 2.16 (1.56, 3.00) | 2.15 (1.54, 3.01) |
| *P* for trend |  |  |  |  | <0.001 | <0.001 | <0.001 | <0.001 |
| **All-cause mortality** |  |  |  |  |  |  |  |  |
| Normal calcium/mildly high phosphorus trajectory | 1810 | 425 | 9150.44 | 46.4 | 1.00 (Ref) | 1.00 (Ref) | 1.00 (Ref) | 1.00 (Ref) |
| Mildly low calcium/moderately high phosphorus trajectory | 1705 | 611 | 8065.62 | 75.8 | 2.08 (1.83, 2.36) | 2.25 (1.97, 2.56) | 1.88 (1.64, 2.16) | 1.88 (1.64, 2.16) |
| Low calcium/high phosphorus trajectory | 722 | 304 | 3493.91 | 87.0 | 3.38 (2.90, 3.95) | 3.54 (3.02, 4.15) | 2.53 (2.12, 3.04) | 2.46 (2.05, 2.96) |
| *P* for trend |  |  |  |  | <0.001 | <0.001 | <0.001 | <0.001 |

Incidence = No. of incident dialysis cases / person-years*1000.

† With competing risk analysis for death

**Model 1:** Adjusted for gender, BMI, smoking status, alcohol consumption, education (n=4210)

**Model 2:** Adjusted for gender, BMI, smoking status, alcohol consumption, education, diabetes, hypertension, cardiovascular disease, primary etiologies of CKD, and baseline eGFR (n=4193)

**Model 3:** Adjusted for gender, BMI, smoking status, alcohol consumption, education, diabetes, hypertension, cardiovascular disease, primary etiologies of CKD, baseline eGFR, and profiles of baseline medication (n=4112)

**Abbreviations:** BMI: body mass index, Ca: calcium, Ca x P: calcium-phosphate product, CI: confidence interval, CKD: chronic kidney disease, eGFR: estimated glomerular filtration rate, ESRD: end stage renal disease, HR: hazard ratio, P: phosphorus

**Supplementary Table 6.** Adjusted hazard ratios (95% confidence interval [CI]) for the risk of progression to end-stage renal disease (ESRD), acute coronary syndrome, and all-cause mortality when further adjusting for the utilization trajectory of calcium-based phosphorus binders, the trajectory of elemental calcium amount from calcium-based phosphorus binders, baseline protein-to-creatinine ratio (PCR), proteinuria, and serum albumin level.

|  | **Model 3** | **Model 3+ Using calcium**  **trajectory** | **Model 3+ Quarterly sum of elemental calcium trajectory** | **Model 3+**  **Urine PCR (mg/g)** | **Model 3+**  **Proteinuria** | **Model 3+**  **Serum albumin (g/dL)** |
| --- | --- | --- | --- | --- | --- | --- |
|  | **Adjusted HR**  **(95% CI)** | **Adjusted HR**  **(95% CI)** | **Adjusted HR**  **(95% CI)** | **Adjusted HR**  **(95% CI)** | **Adjusted HR**  **(95% CI)** | **Adjusted HR**  **(95% CI)** |
| **ESRD requiring dialysis**† |  |  |  |  |  |  |
| Normal calcium/mildly high phosphorus trajectory | 1.00 (Ref) | 1.00 (Ref) | 1.00 (Ref) | 1.00 (Ref) | 1.00 (Ref) | 1.00 (Ref) |
| Mildly low calcium/moderately high phosphorus trajectory | 5.92 (4.71, 7.44) | 4.25 (3.36, 5.38) | 4.33 (3.42, 5.47) | 7.10 (5.22, 9.66) | 6.05 (4.44, 8.23) | 5.54 (4.37, 7.02) |
| Low calcium/high phosphorus trajectory | 15.20 (11.85, 19.50) | 9.81 (7.59, 12.68) | 10.00 (7.73, 12.93) | 19.01 (13.71, 26.35) | 16.33 (11.80, 22.61) | 13.76 (10.64, 17.78) |
| *P* for trend | <0.001 | <0.001 | <0.001 | <0.001 | <0.001 | <0.001 |
| **Acute coronary syndrome**† |  |  |  |  |  |  |
| Normal calcium/mildly high phosphorus trajectory | 1.00 (Ref) | 1.00 (Ref) | 1.00 (Ref) | 1.00 (Ref) | 1.00 (Ref) | 1.00 (Ref) |
| Mildly low calcium/moderately high phosphorus trajectory | 1.94 (1.49, 2.52) | 1.56 (1.18, 2.06) | 1.61 (1.22, 2.12) | 1.98 (1.44, 2.73) | 1.86 (1.34, 2.57) | 1.91 (1.45, 2.51) |
| Low calcium/high phosphorus trajectory | 3.18 (2.30, 4.39) | 2.29 (1.62, 3.25) | 2.39 (1.68, 3.39) | 3.73 (2.55, 5.47) | 3.49 (2.37, 5.14) | 3.13 (2.22, 4.40) |
| *P* for trend | <0.001 | <0.001 | <0.001 | <0.001 | <0.001 | <0.001 |
| **All-cause mortality** |  |  |  |  |  |  |
| Normal calcium/mildly high phosphorus trajectory | 1.00 (Ref) | 1.00 (Ref) | 1.00 (Ref) | 1.00 (Ref) | 1.00 (Ref) | 1.00 (Ref) |
| Mildly low calcium/moderately high phosphorus trajectory | 1.88 (1.64, 2.16) | 1.83 (1.58, 2.12) | 1.85 (1.60, 2.14) | 1.85 (1.56, 2.20) | 1.70 (1.43, 2.02) | 1.78 (1.54, 2.06) |
| Low calcium/high phosphorus trajectory | 2.46 (2.05, 2.96) | 2.39 (1.96, 2.90) | 2.45 (2.02, 2.98) | 2.37 (1.90, 2.95) | 2.20 (1.77, 2.75) | 2.18 (1.80, 2.64) |
| *P* for trend | <0.001 | <0.001 | <0.001 | <0.001 | <0.001 | <0.001 |

Model 3: Adjusted for gender, BMI, smoking status, alcohol consumption, education, diabetes, hypertension, cardiovascular disease, primary etiologies of CKD, baseline eGFR, and profiles of baseline medication (n=4112)

Model 3+Using calcium trajectory (n=4112); Model 3+ Quarterly sum of elemental calcium trajectory (n=4112); Model 3+ Urine PCR (n=3192); Model 3+ Proteinuria (n=3192); Model 3+ serum albumin (n=3788).

**Abbreviations:** BMI: body mass index, CI: confidence interval, CKD: chronic kidney disease, eGFR: estimated glomerular filtration rate, ESRD: end stage renal disease, GBTM: group-based trajectory modelling, HR: hazard ratio, PCR: protein/creatinine ratio.

**Supplementary Figure 1.** Probability of using calcium-based phosphorus binders and quarterly sum of estimated elemental calcium amount (g) from calcium-based phosphorus binders trajectories as defined by group-based trajectory modelling (GBTM). The solid line is the averaged estimated trajectory, whereas the points represent the averaged observed trajectory. (N = 4237). The amount of elemental calcium for calcium carbonate 500 mg and calcium acetate 677 mg is assumed to be 200 mg and 169 mg, respectively.

**
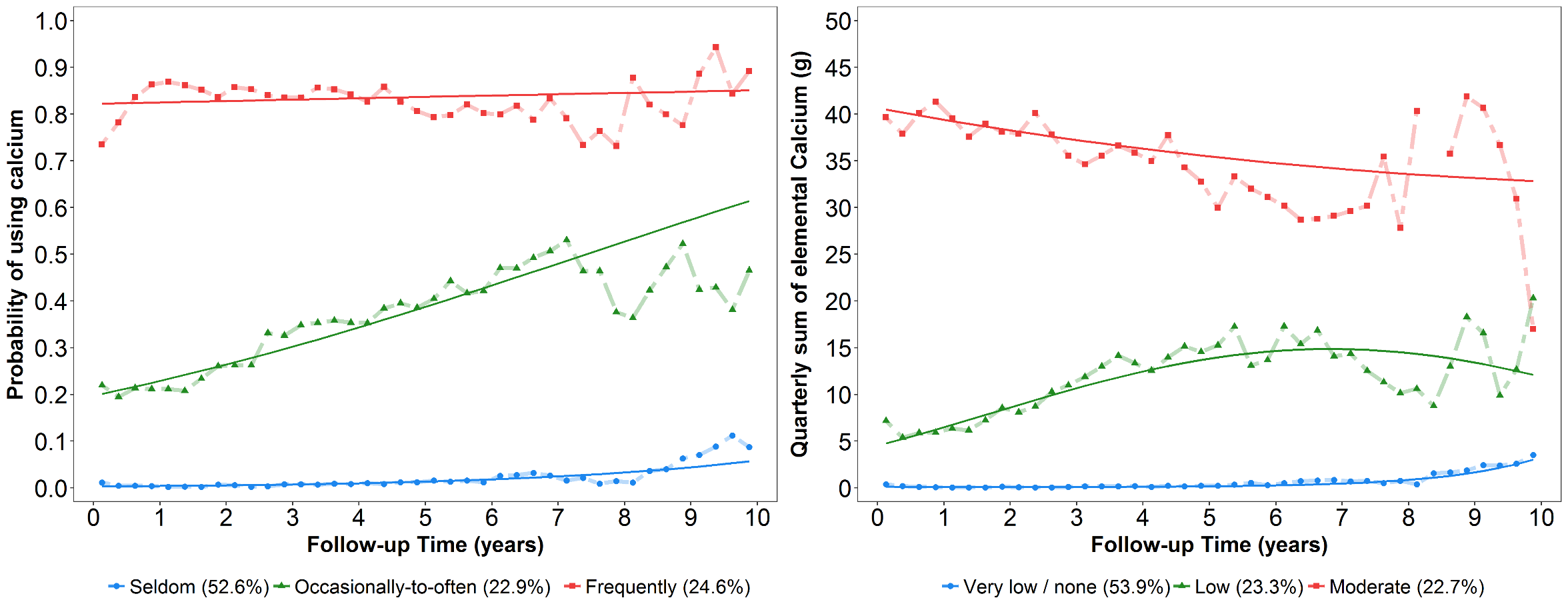
**
